# Pathogenic variants in *CRX* have distinct *cis*-regulatory effects on enhancers and silencers in photoreceptors

**DOI:** 10.1101/gr.278133.123

**Published:** 2024-02

**Authors:** James L. Shepherdson, Ryan Z. Friedman, Yiqiao Zheng, Chi Sun, Inez Y. Oh, David M. Granas, Barak A. Cohen, Shiming Chen, Michael A. White

**Affiliations:** 1 Department of Genetics, Washington University School of Medicine in St. Louis, St. Louis, Missouri 63110, USA;; 2 Edison Family Center for Genome Sciences & Systems Biology, Washington University School of Medicine in St. Louis, St. Louis, Missouri 63110, USA;; 3 Department of Ophthalmology and Visual Sciences, Washington University School of Medicine in St. Louis, St. Louis, Missouri 63110, USA;; 4 Department of Developmental Biology, Washington University School of Medicine in St. Louis, St. Louis, Missouri 63110, USA

## Abstract

Dozens of variants in the gene for the homeodomain transcription factor (TF) cone-rod homeobox (*CRX*) are linked with human blinding diseases that vary in their severity and age of onset. How different variants in this single TF alter its function in ways that lead to a range of phenotypes is unclear. We characterized the effects of human disease-causing variants on CRX *cis*-regulatory function by deploying massively parallel reporter assays (MPRAs) in mouse retina explants carrying knock-ins of two variants, one in the DNA-binding domain (p.R90W) and the other in the transcriptional effector domain (p.E168d2). The degree of reporter gene dysregulation in these mutant *Crx* retinas corresponds with their phenotypic severity. The two variants affect similar sets of enhancers, and p.E168d2 has distinct effects on silencers. *Cis*-regulatory elements (CREs) near cone photoreceptor genes are enriched for silencers that are derepressed in the presence of p.E168d2. Chromatin environments of CRX-bound loci are partially predictive of episomal MPRA activity, and distal elements whose accessibility increases later in retinal development are enriched for CREs with silencer activity. We identified a set of potentially pleiotropic regulatory elements that convert from silencers to enhancers in retinas that lack a functional CRX effector domain. Our findings show that phenotypically distinct variants in different domains of CRX have partially overlapping effects on its *cis*-regulatory function, leading to misregulation of similar sets of enhancers while having a qualitatively different impact on silencers.

The cone-rod homeobox (*CRX*) gene encodes a paired-type homeodomain transcription factor (TF) that is expressed primarily in the retina (Chen et al. 1997; Freund et al. 1997; Furukawa et al. 1997). Animal studies show that *CRX* is essential for the development and maintenance of postmitotic photoreceptors (Furukawa et al. 1999; Muranishi et al. 2011). Variants in *CRX* (OMIM: 602225) have been shown to cause several inherited retinal diseases, including retinitis pigmentosa (RP), cone-rod dystrophy (CoRD), and Leber congenital amaurosis (LCA) (Evans et al. 1994; Swain et al. 1997; Sohocki et al. 1998; Swaroop et al. 1999; Rivolta et al. 2001; Perrault et al. 2003; Ziviello et al. 2005; Nichols et al. 2010; Huang et al. 2012; Koyanagi et al. 2019; Fujinami-Yokokawa et al. 2020; Ng et al. 2020). *CRX* is the only gene implicated in all three of these diseases (Berger et al. 2010; Tran and Chen 2014). Retinopathies associated with *CRX* present with different phenotypes, varying in their cone versus rod predominance, severity, and age of onset. Some *CRX* variants have been shown to cause severe dominant disease, whereas others are pathogenic only in a recessive context (Rivolta et al. 2001; Huang et al. 2012; Hull et al. 2014; Fujinami-Yokokawa et al. 2020; Nishiguchi et al. 2020; Markitantova and Simirskii 2020; Kim et al. 2023).

The variable phenotypes of different pathogenic variants in *CRX* are likely owing to differences in their effects on gene expression (Chen et al. 2002; Tran and Chen 2014). Studies of mouse models carrying the retinopathy-associated variants *Crx*^*R90W*^ or *Crx*^*E168d2*^ show that major changes in gene expression are evident by postnatal day (P) 10, during the critical period of photoreceptor differentiation (Swaroop et al. 2010) and before retinal degeneration can be observed (Tran et al. 2014; Ruzycki et al. 2015; Zheng et al. 2023). These studies also show that the two variants have graded effects on gene expression that correspond with the severity of their retinal phenotypes. Genes affected by *Crx*^*R90W*^ are also misregulated in the presence of the more phenotypically severe *Crx*^*E168d2*^, which affects additional genes. These changes in gene expression are the consequence of prior changes in the activity of possibly thousands of CREs targeted by CRX. Pathogenic variants could lead to misregulation by altering the affinity or kinetics of interactions between CRX and its DNA binding sites, or with cooperatively interacting transcriptional cofactors. The consequences of these disrupted interactions could vary across thousands of different *cis*-regulatory elements (CREs) in ways that depend on their DNA sequence composition. Although the DNA-binding specificity of CRX is well characterized (Corbo et al. 2010; Lee et al. 2010; Chu et al. 2012) and CRX is known to interact with other TFs and transcriptional cofactors (Mitton et al. 2000; Yanagi et al. 2000; Peng et al. 2005; Srinivas et al. 2006; Peng and Chen 2007; Roduit et al. 2009; Hlawatsch et al. 2013; Langouët et al. 2022; Zhuo and Knox 2022; Sun and Chen 2023), changes in *cis*-regulatory activity caused by pathogenic variants are not easily predictable from detailed biochemical knowledge of specific interactions. To understand how pathogenic variants in CRX affect its function as a TF, it is important to consider how these variants alter *cis*-regulatory activity across a broad range of CRX-bound CREs.

Reporter gene assays offer a more direct measure of CRX function at its target CREs than gene expression profiling. We previously used MPRAs in wild-type and *Crx*^−/−^ mouse retinal explants to measure the activity of thousands of genomic and synthetic DNA sequences that are bound by CRX in vivo or that contain CRX-binding motifs (White et al. 2013, 2016; Friedman et al. 2021). These studies revealed important TF motifs and other DNA sequence features that distinguish different classes of CRX-bound CREs, such as enhancers and silencers. Here we sought to understand how two biochemically and phenotypically distinct CRX variants alter its *cis*-regulatory function by characterizing the impact of these variants on the activities of different classes of CRX-bound CREs.

## Results

### Biochemically distinct CRX variants differ in the severity of their effects on CREs

**Figure 1.**
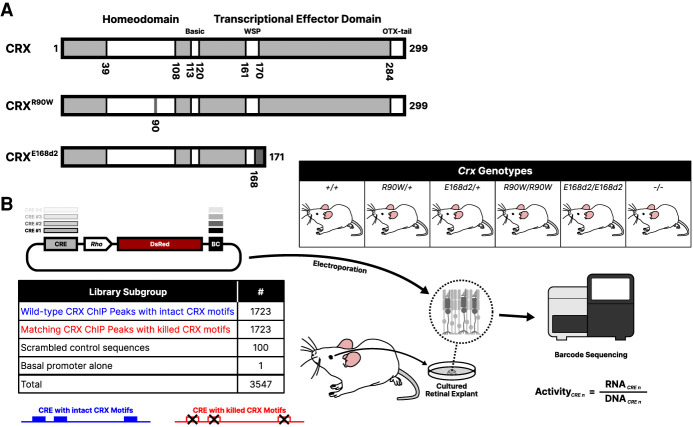
Study overview. (*A*) Schematics of wild-type CRX protein and the p.R90W and p.E168d2 pathogenic variants, containing a point DNA-binding domain mutation or resulting in truncation of the transcriptional effector domain, respectively. (*B*) Outline of the experimental procedure for constructing and testing the CRE libraries. A library of CRE sequences is cloned upstream of a rod photoreceptor-specific *Rhodopsin* (“*Rho*”) minimal promoter driving expression of the DsRed fluorescent protein, with each CRE marked by a unique sequence barcode (BC) in the 3′ UTR. Each library was electroporated into retinal explants with the six indicated *Crx* genotypes. RNA was collected from the retinas, and transcript counts for each element were measured and normalized to abundance in the input DNA library to calculate transcriptional activity.

We tested an MPRA library of CRX-bound CREs in retinal explants from mice that either were wild-type for *Crx* or were carrying prototypical pathogenic variants: the *Crx*^*R90W*^ single-residue substitution variant and the *Crx*^*E168d2*^ frameshift variant (Fig. 1A). Arginine 90 lies in the DNA-binding homeodomain of CRX. The p.R90W variant has been shown to severely weaken CRX binding to target CREs and is reported to cause mild late-onset CoRD or LCA, depending on zygosity (Swaroop et al. 1999; Hull et al. 2014; Tran et al. 2014; Fujinami-Yokokawa et al. 2020; Zheng et al. 2023). Glutamic acid 168 is located within the WSP domain of the transcriptional effector region. The p.E168d2 variant causes a frameshift into the +2 reading frame, which is predicted to lead to a stop codon within three residues. Because this stop codon is located within the final exon, the *Crx*^*E168d2*^ transcript is not predicted to undergo nonsense-mediated decay (Karousis and Mühlemann 2019). The p.E168d2 variant retains the ability to bind DNA but lacks much of the transcriptional effector domain (Chau et al. 2000; Tran et al. 2014). The p.E168d2 variant has been associated with dominant LCA in patients (Freund et al. 1998; Jacobson et al. 1998; Tran et al. 2014). Mice carrying these two variants show significant changes in gene expression by P10, followed by developmental defects and eventual photoreceptor degeneration as early as 3–4 wk of age (Tran et al. 2014; Ruzycki et al. 2015).

To construct an MPRA library, we selected 1723 genomic CRE sequences that are bound by CRX as measured by ChIP-seq in the whole adult mouse retina (Corbo et al. 2010; Ruzycki et al. 2018). CREs were selected to sample CRX-bound CREs from three different chromatin environments that were previously measured at P14 (Ruzycki et al. 2018). To assess the contribution of CRX binding sites to CRE activity, we included a matching CRX motif mutant for every wild-type CRE, in which all copies of the CRX motif were abolished using a mutation known to abrogate TF binding (Fig. 1B; Lee et al. 2010; White et al. 2013). We cloned these 3446 elements in a library with each CRE located upstream of a minimal promoter derived from the endogenous *Rhodopsin* (*Rho*) locus driving expression of the fluorescent protein DsRed (Kwasnieski et al. 2012). Each CRE could be uniquely identified by a short sequence barcode located in the 3′ untranslated region of the *DsRed* transcript. We also included 100 scrambled elements as negative controls, with sequence composition matching that of the average CRE but with no detectable CRX binding motifs as called by FIMO using a *P*-value cutoff of 0.0025 (Grant et al. 2011).

We introduced this library via electroporation into P0 retinal explants from *Crx*^*+/+*^ *C57BL/6J* mice (*+/+*), mice heterozygous or homozygous for *Crx*^*R90W*^ (*R90W/+* and *R90W/W*) or *Crx*^*E168d2*^ (*E168d2/+* and *E168d2/d2*), and *Crx*^−/−^ mice (*−/−*; all on *C57BL/6J* background) (Fig. 1B). After extracting RNA at P8, we sequenced CRE barcodes and normalized read counts to the abundance of each element in the input plasmid library (retina RNA read count/plasmid DNA read count) to compute each element's transcriptional activity. *R*^2^-values between biological replicates were high, ranging from 0.90 in the *+/+* background to about 0.75 in the homozygous mutant backgrounds (Supplemental Fig. S1). We classified each CRE into one of five functional classes based on their transcriptional activity relative to the basal promoter alone: strong enhancer (significant increase in transcriptional activity relative to basal and greater than the 95th percentile of the scrambled sequences), weak enhancer (significant increase in transcriptional activity relative to basal and less than or equal to the 95th percentile of the scrambled sequences), inactive (no significant transcriptional activity difference compared with basal), weak silencer, and strong silencer (using the same criteria and cutoffs as for enhancers, but with significantly less transcriptional activity than basal) (Friedman et al. 2021).

**Figure 2.**
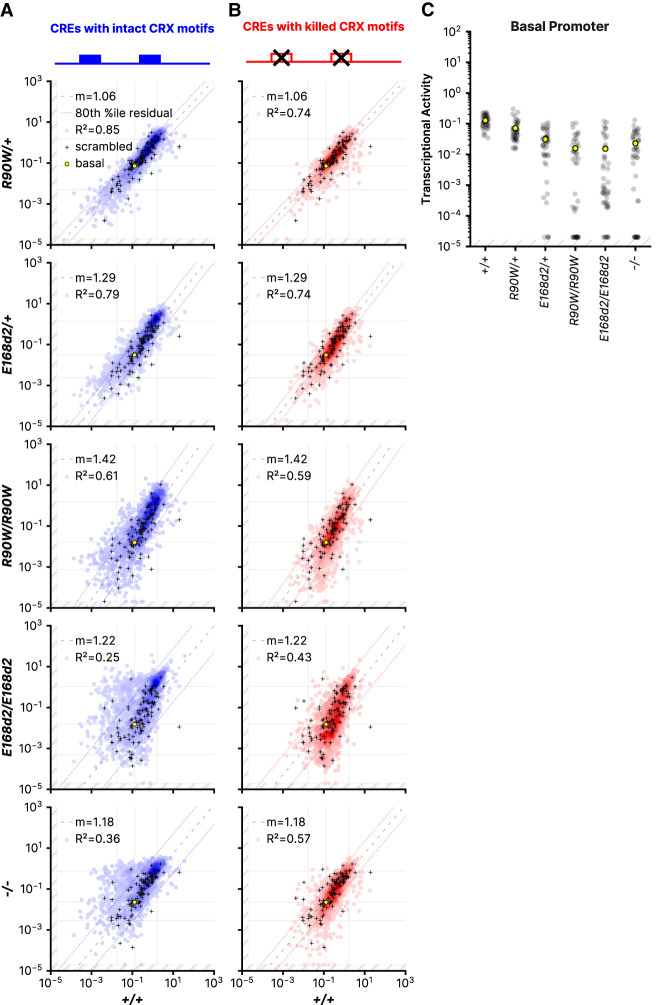
CRE activity in retinas with different *Crx* genotypes. (*A*,*B*) Correlations of the transcriptional activity of each CRE with intact CRX motifs (*A*) or CRE with killed CRX motifs (*B*) in the indicated genotypes. Superimposed black cross symbols indicate the transcriptional activity of the 100 scrambled control sequences, and a yellow circle shows the activity of the basal *Rho* promoter alone. Using the scrambled control sequences to establish an empirical null distribution of CRE activity, we performed robust regression using a trimmed mean M-estimator (fit plotted as a dashed line) for each genotype comparison. The *outer* dotted lines indicate the 80th percentile of the residuals of the library CREs relative to the regression of the scrambled sequences. The *outer* vertical and horizontal lines indicate the thresholds for the “strong silencer” and “strong enhancer” classes, and the *inner* horizontal and vertical lines correspond to the basal *Rho* promoter alone. (*C*) Activity of the *Rho* basal promoter construct in each of the indicated genotypes. Each black dot indicates a different unique sequence barcode; the mean across barcodes is shown as a yellow dot. In all panels, transcriptional activity was adjusted for visualization purposes using a fixed pseudocount of 2 × 10^−5^; the out-of-bound region is indicated by the light gray stripes.

We first evaluated the global *cis*-regulatory effects of the variants by comparing CRE expression from each mutant mouse line to retinas from *+/+* mice (Fig. 2A,B). We observed an increasingly pronounced global dysregulation of CRE activity that correlated with increasing phenotypic severity. Phenotypic severity was previously shown to correspond with the identity and zygosity of the mutants (Tran et al. 2014). Little retinal impairment is observed in *R90W/+* mice, whereas progressive rod dystrophy occurs in *E168d2/+* mice. Unlike the heterozygotes, all homozygous mutant mice are blind, with *E168d2/d2* mice showing the most severe photoreceptor degeneration among the homozygous mutant mice. Consistent with the relative phenotypic severities of the genotypes, we found that CRE activity in *R90W/+* retinas was tightly correlated with activity in *+/+* retinas (*R*^2^ = 0.85). In contrast, CRE activity in *E168d2/d2* retinas correlated poorly with matched *+/+* measurements (*R*^2^ = 0.25).

The *Rho* minimal promoter contains CRX binding sites, and thus its intrinsic transcriptional activity is also altered by CRX variants. We quantified this effect by calculating the regression slope for each mutant genotype compared with the wild type, using a trimmed mean M-estimator based on the 100 scrambled control sequences (Fig. 2A, M-estimator fit is plotted by the dashed line). The slope increasingly deviates from one in the more severe genotypes, indicating that a decline in basal promoter activity contributes to the dysregulation of the reporter genes. The M-estimator values are consistent with an observed decrease in the activity of the *Rho* minimal promoter alone, as measured by MPRA barcodes that tag the basal promoter (Fig. 2C). Together, the *R*^*2*^-value and the M-estimator value show that both the degree of global CRE dysregulation and the reduction in basal promoter levels correspond with phenotypic severity. Abolishing CRX motifs improves the correlation between the wild type and the *E168d2/d2* and *−/−* genotypes (Fig. 2B), indicating that CRE dysregulation in the mutant genotypes is mediated by CRX motifs. Consistent with the fact that the assayed CREs are taken from CRX-bound loci, most alter their activity when CRX motifs are abolished (Supplemental Fig. S2). These results show that both the *Crx* genotype and the presence of CRX binding motifs affect CRE activity, and thus, they indicate that CRE dysregulation is a direct consequence of altered CRX function.

We observed graded changes in the activity of strong and weak enhancers in the mutant genotypes. In *R90W/+* retinas, 134 (13%) strong and weak enhancers lost substantial activity, defined as a reduction to a lower activity class (Supplemental Fig. S3A). Additional enhancers lost activity in the *R90W/W* (n = 164, 16%) and *E168d2/d2* genotypes (n = 212, 21%). A subset of weak enhancers showed amplified transcriptional activity relative to the basal promoter in the mutant genotypes. One hundred fourteen weak enhancers (13%) increased activity relative to the *Rho* minimal promoter in *R90W/+* retinas, whereas 150 (18%) and 195 (22.9%) were amplified in *R90W/W* and *E168d2/d2* retinas, respectively (Supplemental Fig. S3A). This increase relative to basal levels was not owing to an absolute increase in transcriptional activity but was instead owing to a decrease in basal activity in the more severely affected genotypes (Fig. 2A,C). This effect indicates that some CREs retained the ability to drive transcriptional activity despite the loss of basal promoter activity in the mutant genotypes, possibly owing to the presence of binding sites for TFs other than CRX. However, we searched for TF binding motifs enriched in different subsets of enhancers that gained or lost activity and found none that were strongly associated with a specific pattern of response to the mutant genotypes. This suggests that changes in activity common to subsets of enhancers are not defined by the presence or absence of common motifs but instead by a more complex *cis*-regulatory grammar defined by multiple interacting TFs.

The most striking difference between the p.R90W and p.E168d2 variants was in their effect on silencers, particularly in the homozygous genotypes. Although some silencers were moderately derepressed in *R90W/W* retinas, we observed a more extensive loss of repression among silencers in *E168d2/d2* and *−/−* retinas, both of which lack the CRX transcriptional effector domain (Fig. 2A). The extensive derepression observed in *E168d2/d2* retinas relative to *R90W/W* retinas is consistent with results from a prior RNA-seq analysis of these mouse models (Ruzycki et al. 2015). In this study, a larger fraction of differentially expressed genes were up-regulated in *E168d2/d2* retinas (248 of 673 differentially expressed genes, 37%) compared with *R90W/W* retinas (70 of 265 differentially expressed genes, 26%, *P* = 0.003, Fisher's exact test). By RNA-seq analysis alone, gene up-regulation could be an indirect effect of loss of CRX function, possibly owing to changes in the levels of other TFs. However, our finding that silencers are derepressed in *E168d2/d2* retinas suggests that gene up-regulation is a direct effect caused by the loss of CRX-mediated silencing.

### Silencers are enriched among CREs near cone photoreceptor genes

A previous RNA-seq analysis in whole mouse retinas found that genes enriched in rod photoreceptors were largely down-regulated in the presence of the p.R90W and p.E168d2 variants, whereas genes enriched in cone photoreceptors were often up-regulated (Ruzycki et al. 2015). Because 80% of the cells in mouse retina are rod photoreceptors (Jeon et al. 1998), gene expression analyses of whole retina primarily capture effects that occur in rods. Thus the up-regulation of cone genes and the down-regulation rod genes in whole retina analyses reflect changes occurring in rod photoreceptors in the *Crx* mutant genotypes.

**Figure 3.**
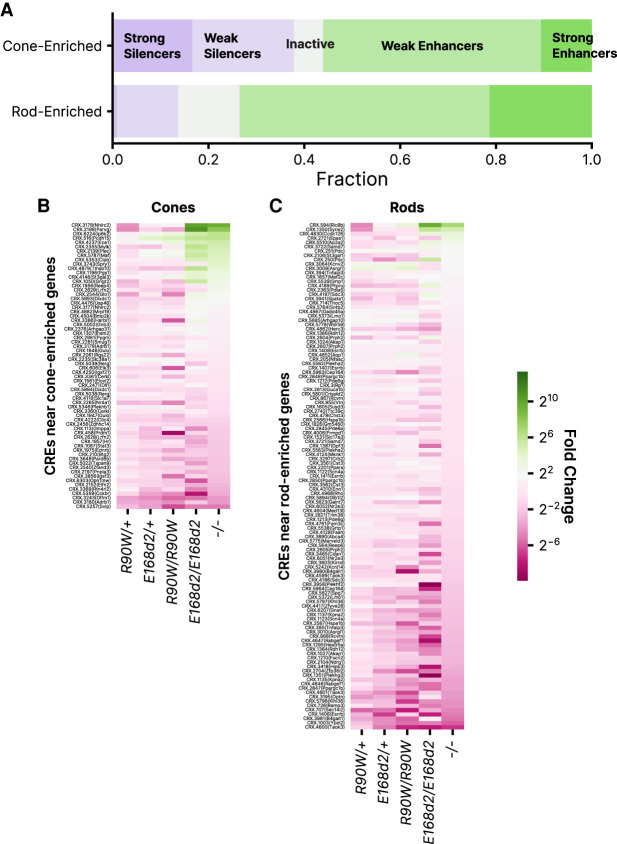
Activities of CREs near genes enriched in rod or cone photoreceptors. (*A*) Distribution of activity classes in *+/+* retina for 66 and 117 CREs within 100 kbp of cone- or rod-enriched genes, respectively, as defined by Ruzycki et al. (2015). (*B*) Fold change relative to *+/+* of 66 CREs near cone-enriched genes. (*C*) Fold change relative to *+/+* of 117 CREs near rod-enriched genes. In *B* and *C*, rows are sorted by fold change in *−/−*.

We examined whether changes in CRE activity in the mutant genotypes paralleled the previously measured changes in rod and cone gene expression. Although CREs included in the MPRA library were not chosen for their proximity to known photoreceptor genes, we nonetheless identified 183 CREs in the library within 100 kbp of a transcription start site (TSS) of 241 previously identified rod- or cone-enriched genes. This included 117 CREs near rod genes and 66 near cone genes. We found that CREs near cone genes were enriched for silencers compared with CREs near rod genes (37.8% vs. 13.7%), whereas rod genes were enriched for enhancers (65.0% vs. 56.1%, *P* = 0.022, chi-square test for independence) (Fig. 3A). Correspondingly, CREs near cone genes were more likely to be derepressed in the mutant genotypes, particularly in *E168d2/d2* and *−/−* retinas (Fig. 3B,C). Although changes in the expression of a gene are the cumulative result of changes in the activity of multiple CREs, these results show that an episomal assay of CRE activity partially recapitulates changes in gene expression. CRX has the ability to repress cone genes in cooperation with other TFs such as NR2E3 (Peng et al. 2005). Our results suggest that this repressive role requires the CRX effector domain and that the loss of repressive activity of the p.E168d2 variant may explain its more severe phenotype.

### A subset of CREs convert from silencers to enhancers when the CRX effector domain is absent

**Figure 4.**
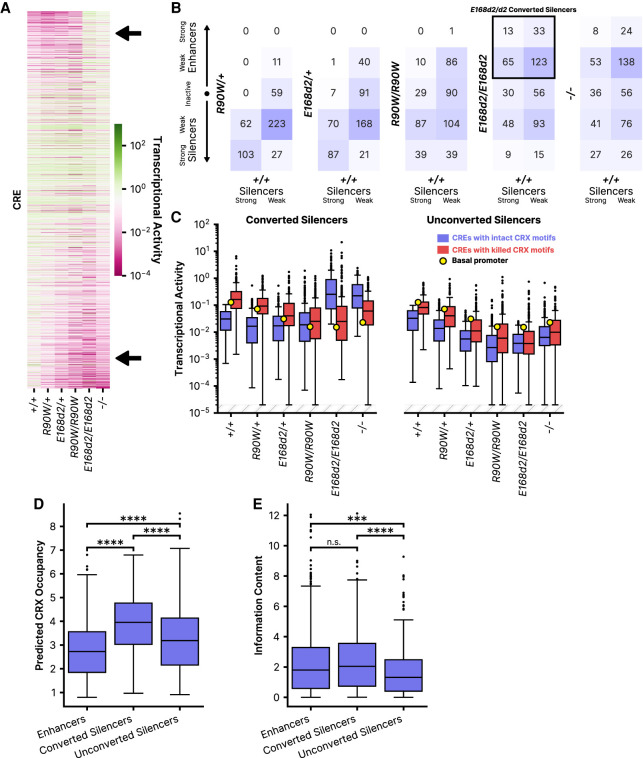
Identification and characterization of silencers that convert to enhancers in *Crx* mutant genotypes. (*A*) Heatmap comparing the transcriptional activity of each CRE across the six *Crx* genotypes. In the *top right* (*upper* arrow), note a group of CREs with generally low activity in all but the *E168d2/d2* and *−/−* genotypes. Along the *bottom* (*lower* arrow), note the stepwise reduction in activity with increasing phenotypic severity. CREs are sorted by the ratio of transcriptional activity in the *+/+* and *−/−* genotypes. (*B*) Classifications across genotypes for CREs classified as strong silencers (*left* column of each heatmap) or weak silencers (*right* column) in wild-type retinas. (*C*) Transcriptional activity of wild-type or motif mutant CREs across genotypes for 234 silencers in *+/+* that convert to enhancers in *E168d2/d2* (converted), or the remaining 251 silencers in *+/+* that do not convert to enhancers in *E168d2/d2* (unconverted). A yellow dot indicates the transcriptional activity of the basal promoter alone in each genotype. (*D*) Predicted CRX occupancy of the same silencer subgroups as in panel *C* and CREs that act as enhancers in *+/+*. (*E*) Information content of the same subgroups as panel *D*. (*) *P* < 0.05, (**) *P* < 0.01, (****) *P* < 0.0001 via two-sided Mann–Whitney–Wilcoxon test. In panel *C*, transcriptional activity was adjusted for visualization purposes using a fixed pseudocount of 2 × 10^−5^; the out-of-bound region is indicated by the light gray stripes.

Most CREs show a graded response across the *Crx* mutants, with the effects differing primarily in their severity (Fig. 4A). However, a subset of silencers not only lose repressive activity but also gain enhancer activity in *E168d2/d2* and *−/−* retinas (Fig. 4A, upper arrow). We identified 234 sequences classified as weak or strong silencers in the wild type that become weak or strong enhancers in *E168d2/d2* and *−/−* retinas (Fig. 4B, outlined by the black square; Supplemental Fig. S3A,C). There is a corresponding change in the function of CRX motifs in these CREs (Fig. 4C; Supplemental Fig. S3B). Mutating CRX motifs in this subset of silencers causes a loss of repression in genotypes with an intact CRX transcriptional effector domain (*+/+*, *R90W/+*, *E168d2/+*, *R90W/W*), whereas the same motif mutations cause loss of activation in genotypes that lack the CRX transcriptional effector domain (*E168d2/d2*, *−/−*). This effect does not occur in silencers that are merely derepressed and that do not convert to enhancers in *E168d2/d2* and *−/−* genotypes (Fig. 4C). These results show that some CRX motifs are pleiotropic, mediating repression in the presence of wild-type CRX (when these CREs act as silencers) and mediating activation when the CRX transcriptional effector domain is lost in the *E168d2/d2* and *−/−* genotypes (when these CREs act as enhancers). Because this effect occurs in *−/−* retinas that lack CRX entirely, these pleiotropic sites must be bound by another TF in the absence of CRX. That TF is possibly the CRX homolog OTX2, which is also expressed in developing photoreceptors and a has a similar DNA-binding specificity (Koike et al. 2007; Chu et al. 2012).

To further characterize CREs that convert from silencers to enhancers, we examined two sequence features that we previously showed were characteristic of CRX-bound enhancers or CRX-bound silencers (Friedman et al. 2021). Compared with enhancers, CRX-bound silencers tend to contain more copies of the CRX motif, which we quantify using a threshold-free motif scan called CRX predicted occupancy (White et al. 2013, 2016; Friedman et al. 2021). In contrast to silencers, enhancers are characterized by the presence of a more diverse collection of motifs for eight other lineage-specific TFs. We quantify motif diversity with an “information content” score, based on Boltzmann entropy, that considers the number and identity of motifs in a CRE. Enhancers have higher information content than do silencers (Friedman et al. 2021). Information content is determined by motifs or motif families for CRX/OTX2, NRL (leucine zipper), NEUROD1 (E-box binding factor), RORB (nuclear receptor), MAZ/SP4 (C2H2 zinc finger), MEF2D (MADS-box), RAX (Q50 homeodomain), and GFI1 (C2H2 zinc finger repressor). In sum, prior analyses show that enhancers are characterized by high information content and modest CRX predicted occupancy, whereas silencers are characterized by low information content and high CRX predicted occupancy (Friedman et al. 2021).

Using CRX predicted occupancy and information content scores, we found that CREs that convert from silencers to enhancers in *E168d2/d2* and *−/−* retinas have features of both silencers and enhancers. Most silencers that convert to enhancers have higher predicted CRX occupancy than do wild-type enhancers (Fig. 4D), which is typical of CRX-bound silencers (Friedman et al. 2021). Additionally, most silencers that convert to enhancers have an even higher CRX predicted occupancy than that of other silencers (median, 3.95 vs. 3.19) (Fig. 4D). Yet unlike other silencers, most silencers that convert to enhancers also have the high information content that is characteristic of enhancers (Fig. 4E). The information content of this group is nearly as high as the information content of CREs that act as enhancers in wild-type retinas (median, 2.05 vs. 1.80). The mixed sequence characteristics of these CREs may explain why they convert from silencers to enhancers in *E168d2/d2* and *−/−* retinas. They function as silencers in wild-type retinas owing to their high CRX occupancy. Upon loss of the CRX transcriptional effector domain, they cease to function as silencers, possibly owing to the loss of interactions with a CRX-interacting repressor complex. However, their high information content, reflecting the presence of motifs for other TFs, enables these CREs to function as enhancers in *E168d2/d2* and *−/−* retinas, in contrast to low-information content silencers that simply lose their repressive activity. The additional TFs recruited by the diverse binding motifs in these CREs may interact with OTX2 or some other TF that binds the pleiotropic CRX motifs in the *E168d2/d2* and *−/−* genotypes.

We searched for additional enriched motifs in these potentially bifunctional CREs using simple enrichment analysis (Bailey and Grant 2021). We found that CREs that convert from silencers to enhancers are enriched in motifs for a variety of paired-type homeodomain TFs. CRX and its homolog OTX2 are K50 paired-type homeodomains that contain a lysine at residue 50 of the DNA-binding domain, which determines the specificity of these TFs for a TAATCC consensus sequence (Furukawa et al. 1997; Chu et al. 2012). Although CRX and OTX2 play critical roles in photoreceptors and bipolar cells, additional non-K50 paired-type homeodomain TFs are active in several retinal cell types (Hennig et al. 2008; Zagozewski et al. 2014). Silencers that convert to enhancers are enriched in motifs for some of these other homeodomain TFs: LHX-family TFs (horizontal and Mueller glial cells, *P* = 2.02 × 10^−8^), VSX1 and VSX2 (cone bipolar cells and retinal ganglion cells, *P* = 1.23 × 10^−5^), ISL2 (retinal ganglion cells, *P* = 2.87 × 10^−6^), VAX1 and other NK-family TFs (role in ventral retina dorsalization, *P* = 1.78 × 10^−5^), and RAX (photoreceptors and bipolar cells, *P* = 2.13 × 10^−4^). These CREs are also strongly enriched in homeodomain dimer motifs. Compared with CREs that remain enhancers and silencers in both wild-type and *E168d2/d2* retinas, CREs that convert from silencers to enhancers show a 1.5-fold enrichment of homeodomain motif dimers (71.2% of bifunctional CREs contain at least one dimer vs. 47.4% among CREs that remain silencers or enhancers, *P* = 1.78 × 1^−10^). Paired-type homeodomain TFs bind as both homo- and hetero-dimers, and the presence of these dimers in potentially bifunctional CREs may enable these elements to play regulatory roles in multiple cell types (Wilson et al. 1993; Rister et al. 2015; Hughes et al. 2018).

Finally, we asked whether potentially bifunctional CREs are cobound by the corepressor BCOR, a Polycomb repressive complex 1 (PRC1) factor that cobinds with CRX and is linked with early-onset retinal degeneration (Langouët et al. 2022). Only a minority of all silencers in wild-type retinas overlapped a BCOR binding site (11.8%). However, silencers that convert to enhancers in *E168d2/d2* and *−/−* retinas were even less likely to be bound at P0 by BCOR (4.7%, *P* = 7.2 × 10^−4^, chi-square test). This suggests that these potentially bifunctional elements are distinct from other silencers and that their silencing may not depend on PRC1.

### Chromatin environments of CRX-bound sequences are partially predictive of episomal MPRA activity

CREs in the MPRA library were selected from different genomic chromatin environments. All library CREs were selected from sites of accessible chromatin (measured by ATAC-seq at P14) (Ruzycki et al. 2018) that overlapped CRX ChIP-seq peaks (measured in adult retina) (Corbo et al. 2010). These sites varied in other chromatin annotations (Ruzycki et al. 2018). CRX-bound accessible sites were classified into four groups based on these additional annotations (Table 1; Ruzycki et al. 2018): (1) the presence or absence of histone marks H3K4me3 and H3K27ac in P14 wild-type retinas and (2) the loss or retention of chromatin accessibility in P14 *−/−* retinas (i.e., whether accessibility is CRX dependent or independent). For each group, the typical developmental timing of the onset of chromatin accessibility was assigned using a DNase I hypersensitivity analysis (Wilken et al. 2015). Group A sequences are positive for the promoter-associated H3K4me3 epigenetic mark (Vermeulen et al. 2010) and H3K27ac, a mark associated with active CREs (Creyghton et al. 2010), and they are largely are proximal (within 5 kbp) to a TSS. Group C is defined by the presence of H3K27ac without H3K4me3 and largely includes distal CREs (within 5–500 kbp of a TSS). Group D sites lack both H3K4me3 and H3K27ac, indicating that they are inactive at P14 despite being accessible. These sites show evidence of late activation at 8 wk and are also largely distal to a TSS. Group B sites, defined by the presence of H3K4me3 and the absence of H3K27ac, were not included in our MPRA library because this group includes fewer than 3% of P14 ATAC-seq peaks and likely does not represent a biologically relevant combination of chromatin annotations. Within each group, CRX-bound sites show either CRX-dependent or CRX-independent chromatin accessibility. CRX-independent Group A sequences are open in multiple nonretinal cell types and are accessible in the retina throughout postnatal development, whereas all other subgroups are primarily accessible only in the retina and vary in the developmental timing of their accessibility (Table 1).

**Table 1.** Features of chromatin environments of MPRA library sequences

Group	Histone marks		Typical distance to TSS	CRX-dependent accessibility	Timing and specificity of accessibility	No. of CREs in MPRA library
	H3K4me3	H3K27ac				
A	+	+	0–5 kb	Independent	Open in other cell types, always accessible	249
				Dependent	Retina-specific, late accessibility	632
C	−	+	5–500 kb	Independent	Retina-specific, early accessibility	311
				Dependent	Retina-specific, late accessibility	366
D	−	−	5–500 kb	Independent	Retina-specific, intermediate accessibility	76
				Dependent	Retina-specific, early accessibility	89

**Figure 5.**
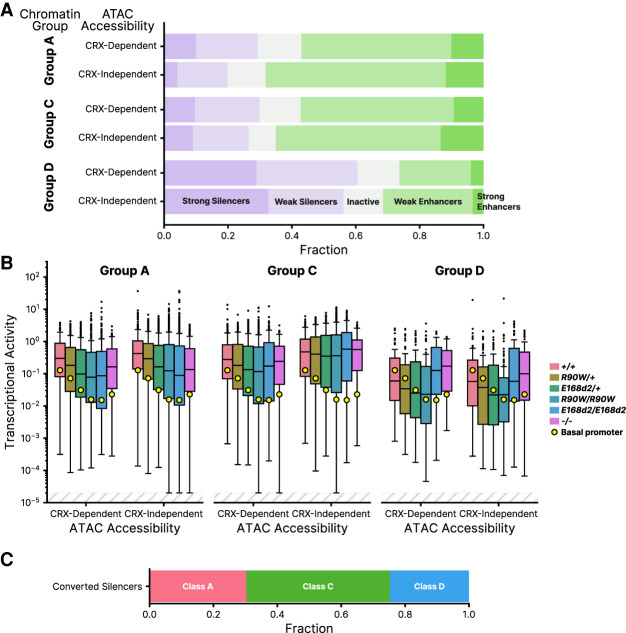
CRE activities analyzed by chromatin annotations at sites of genomic origin. (*A*) Distribution of CRE activity classes in *+/+* retina separated into groups based on chromatin annotations of the site of genomic origin and CRX-dependent ATAC accessibility (Ruzycki et al. 2018). (Group A) H3K4me3^+^ H3K27ac^+^, (Group C) H3K4me3^−^ H3K27ac^+^, (Group D) H3K4me3^−^ H3K27ac^−^. (*B*) Transcriptional activity of all CREs, separated by chromatin and ATAC group, across all genotypes. (*C*) Chromatin classification breakdown of *E168d2/d2* converted silencers (elements classified as weak or strong silencers in *+/+* that become weak or strong enhancers in *E168d2/d2*). In panel *B*, transcriptional activity was adjusted for visualization purposes using a fixed pseudocount of 2 × 10^−5^; the out-of-bound region is indicated by the light gray stripes.

We found that CREs taken from different chromatin environments showed systematic differences in episomal MPRA activity in P8 wild-type retinas. CRX-independent Group A CREs contain the highest proportion of enhancers, contain the lowest proportion of silencers, and show, on average, the highest MPRA activity (Fig. 5A,B). This contrasts with CRX-dependent Group A CREs, which include fewer enhancers and more silencers and show significantly lower average MPRA activity (mean log_2_ RNA/DNA = −1.083 vs. −0.477 for dependent vs. independent Group A, *P* = 5.0 × 10^−4^, independent two-sample *t*-test) (Fig. 5A,B). The difference in the proportion of enhancers and silencers in the two Group A subclasses corresponds with the developmental timing of their chromatin accessibility. CRX-independent Group A CREs are fully accessible by P1 and are more likely to be MPRA enhancers at P8. Group CRX-dependent CREs first become accessible around P7 and reach peak accessibility later in development, and they drive lower transcriptional activity, on average, in the MPRA at P8.

A similar trend was observed for the distal regulatory elements of Group C, which become accessible later in development. In Group C, CRX-independent CREs are partially accessible at P1 and almost fully accessible at P7, whereas CRX-dependent CREs only become partially accessible at P7 and reach full accessibility by 8 wk. Consistent with these differences in the developmental timing of chromatin accessibility in Group C, CRX-independent CREs (intermediate accessibility) have somewhat higher average MPRA activity than do CRX-dependent CREs (late accessibility; mean log_2_ RNA/DNA = −1.101 vs. −0.672, *P* = 0.028, independent two-sample *t*-test) (Fig. 5B). The majority of Group D CREs, which lack the H3K27ac mark of active regulatory elements, are silencers and inactive sequences, and they show the lowest levels of MPRA activity of all chromatin environment groups (Fig. 5A,B). We also examined the distribution of converted silencers among the chromatin groups and found that they are largely distal elements from Groups C and D (Fig. 5C; Supplemental Fig. S3D).

The correspondence between episomal MPRA activity and features of the genomic chromatin environment suggests that the sequence-encoded features responsible for MPRA activity also drive key chromatin changes at native genomic loci over the course of retinal development. The increased fraction of silencers among regulatory elements that become accessible later in development suggests that these CREs may be repressive early in development, preventing premature expression of late developmental genes. The extensive derepression of silencers in the *E168d2/d2* and *−/−* mouse models suggests that the greater severity of these genotypes may be caused in part by premature activation of distal CREs and a consequent disruption of developmentally timed gene expression.

### CRX variants have no systematic effects on cooperativity between CRX binding sites

We designed the MPRA library to test whether CRX variants systematically affected cooperative interactions between CRX binding sites. The library included 100 CREs with exactly two CRX binding motifs for which we designed single- and double-CRX-motif mutants. If the two CRX motifs in a CRE act cooperatively, the sum of the effects of the single-motif mutants will exceed the effect of the double-motif mutant. We computed the sum of the effects of the single-motif mutants on MPRA activity and divided that sum by the effect of the double-motif mutant. A ratio greater than one indicated that the two motifs act cooperatively, whereas a ratio near one indicates no cooperativity. A ratio less than one could indicate either redundancy or anticooperativity between the two CRX motifs. We found that very few of the 100 tested CREs showed evidence of cooperativity between CRX sites, and none of the mutant *Crx* genotypes systematically increased or decreased cooperativity (Supplemental Fig. S4). Although the p.R90W and p.E168d2 variants may affect cooperative interactions between CRX and other TFs or may affect CRX–CRX cooperativity in a very specific contexts, we found no systematic effects on homotypic cooperative interactions between CRX molecules.

## Discussion

We used MPRAs conducted in P0–P8 whole-retinal explants to understand how two biochemically distinct pathogenic variants alter the *cis*-regulatory function of CRX. In both humans and mouse models, pathogenic CRX variants often lead to progressive photoreceptor degeneration beginning in young adulthood, but evidence from mouse models indicates that misregulated gene expression can be observed much earlier (Tran et al. 2014; Ruzycki et al. 2015; Zheng et al. 2023). In this study, we assayed CRX function when changes in *cis*-regulatory activity are already evident but before photoreceptor degeneration has begun (Tran et al. 2014). All CREs included in the MPRA library were selected from genomic loci that are at least partially accessible at P14 and bound by CRX in adult retina (Corbo et al. 2010; Ruzycki et al. 2018), but they were sampled from a variety of chromatin environments, and they vary in the developmental timing of their activation (Wilken et al. 2015; Ruzycki et al. 2018). Thus, our MPRA study was designed to test CRX function across a broad range of its target CREs, during the time of early postmitotic photoreceptor differentiation in mouse models.

Consistent with a prior RNA-seq analysis of the same mouse models (Ruzycki et al. 2015), we observed graded degrees of global dysregulation in CRE activity that correspond with the severity of the retinal phenotypes (Figs. 2A, 4A). Our results suggest that the phenotypic differences between mice expressing the p.R90W and p.E168d2 variants can be partially explained by the fact that p.E168d2 disrupts CRX *cis*-regulatory function at more CREs, particularly silencers. Although p.R90W and p.E168d2 affect largely overlapping sets of wild-type enhancers (Fig. 4A, lower arrow), p.E168d2 causes extensive silencer derepression that does not occur with p.R90W (Fig. 4A, upper arrow). Silencer derepression also occurs in *−/−* retina, showing that the repressive activity of silencers requires the presence of the CRX transcriptional effector domain, which has been classically considered an activation domain (Chen et al. 1997; Peng and Chen 2007). However, a recent study found that the transcriptional effector domain of human CRX is capable of both activation and repression and that homeodomain TFs are enriched in such bifunctional effector domains (DelRosso et al. 2023). The ability of CRX to act as both a repressor and an activator is consistent with its role as a regulator of distinct gene expression programs in multiple retinal cell types (Swaroop et al. 2010).

A loss of repression of cone photoreceptor genes was observed by RNA-seq in whole *E168d2/d2* mouse retina (Ruzycki et al. 2015). Consistent with this result, we found that CREs near cone photoreceptor genes were disproportionately silencers in our episomal MPRA and that these silencers were derepressed in the *E168d2/d2* and *−/−* genotypes (Fig. 3). RNA-seq and MPRA analyses performed in whole retina largely reflect *cis*-regulatory activity in rod photoreceptors, because ∼80% of the cells of mouse retinas are rods (Jeon et al. 1998) and rods make up 87% of plasmid-containing cells in a whole-retina MPRA (Kwasnieski et al. 2012; Zhao et al. 2023). Taken together, RNA-seq and MPRA results suggest that the loss of the CRX transcriptional effector domain leads to inappropriate activation of cone genes in rod photoreceptors early in development and that this is a direct consequence of altered CRX function at its target silencers. Variants like p.E168d2 that truncate the CRX effector domain may abolish its ability to interact, directly or indirectly, with other factors required for repression, such as NR2E3 and SAMD7 (Peng et al. 2005; Hlawatsch et al. 2013; Omori et al. 2017), and thereby impair rod differentiation.

**Figure 6.**
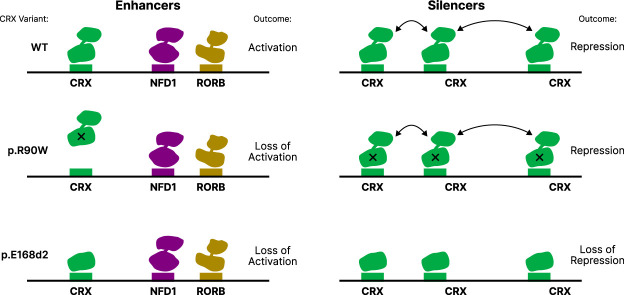
A model of differential enhancer and silencer sensitivity to CRX mutants. Silencers, with higher predicted CRX occupancy, engage in effector domain–mediated homotypic interactions that permit CRX to remain bound even when the DBD is weakened by the p.R90W variant. The p.E168d2 variant lacks most of the effector domain and cannot engage in these interactions. Enhancers have lower CRX predicted occupancy and engage in fewer homotypic interactions.

Results of prior MPRA studies show that whether CRX acts as an activator or repressor at a particular CRE depends on local motif composition (White et al. 2013, 2016; Hughes et al. 2018; Friedman et al. 2021; Loell et al. 2024). We previously reported that CRX-bound silencers tend to have more copies of the CRX motif than enhancers do, whereas enhancers contain more motifs for other photoreceptor TFs (White et al. 2016; Friedman et al. 2021). These features of the sequence composition of enhancers and silencers suggest a model that may explain why enhancers and silencers respond differently to the p.R90W and p.E168d2 variants (Fig. 6). At enhancers, which have moderate CRX occupancy, both variants cause a loss of active CRX, either by reduction in DNA binding (p.R90W) or by loss of the effector domain (p.E168d2). At silencers, the presence of more copies of the CRX motif results in greater affinity for CRX, which makes silencers less sensitive to the reduced DNA binding affinity of p.R90W. High CRX occupancy at silencers might also be promoted by homotypic interactions mediated by the transcriptional effector domain, which our results show is necessary for repression at silencers. Although the biophysical nature of these homotypic interactions is unknown, there is clear evidence that the presence of multiple copies of the CRX motif often leads to repression in both genomic and synthetic CREs (White et al. 2016; Friedman et al. 2021; Loell et al. 2024).

Characteristic sequence features of both silencers and enhancers are present in the potentially bifunctional CREs that we report here. These CREs, which are silencers in *+/+* retinas and convert to enhancers in *E168d2/d2* and *−/−* retinas, have especially high copy numbers of the CRX motif, as well as motifs for other lineage-specific TFs (Fig. 4D,E). The ability of these silencers to convert to enhancers in genotypes that lack the CRX transcriptional effector domain may indicate that, in a wild-type context, they play a role as enhancers in other cell types or at other developmental time points. The CRX binding sites in these CREs are clearly pleiotropic, being necessary for repression in *+/+* retinas and for activation in *E168d2/d2* and *−/−* retinas. The activity of these sites in *−/−* retinas suggests that they are reused by another TF in cellular contexts in which CRX levels are reduced or absent and that this other TF activates at binding sites where CRX represses (White et al. 2016). A probable candidate TF is the CRX homolog OTX2, which plays a major role in retinal development and whose binding specificity is nearly identical to that of CRX (Furukawa et al. 2002; Nishida et al. 2003; Koike et al. 2007; Chu et al. 2012). These bifunctional CREs thus may be CRX-bound silencers in rod photoreceptors (where CRX expression is high and OTX2 expression is low) and OTX2-bound enhancers in another cell type with high OTX2 and low CRX expression, such as bipolar cells (Koike et al. 2007; Murphy et al. 2019). CRX and OTX2, despite similar DNA binding specificity, have nonoverlapping functions (Terrell et al. 2012; Yamamoto et al. 2020), which would allow them to play different regulatory roles at the same CREs. Currently, there are a limited number of examples of CREs that act as silencers in one cell type and enhancers in another (Gisselbrecht et al. 2020). However, such bifunctional elements could be part of an effective regulatory strategy during the development of a population of multipotent progenitor cells into closely related cell types with overlapping regulators, as occurs in the retina (Swaroop et al. 2010; Murphy et al. 2019).

Our results show that the activities of CREs measured episomally by MPRA at P8 show some correspondence with features of the chromatin environment of their native genomic loci. Distal CREs whose chromatin accessibility does not peak until 8 wk after birth were depleted of strong enhancers and enriched for silencers, whereas proximal CREs that are already accessible by P0 contained the highest fraction of strong enhancers of any chromatin group. The enrichment of silencers among late-acting CRX-bound CREs suggests that these elements may have sequence-encoded repressive features that prevent premature activation. Our results indicate that episomal MPRAs capture some sequence-encoded features that determine differences in chromatin environment in the genome.

MPRAs offer the ability to characterize how pathogenic coding variants in TFs affect the *cis*-regulatory activity of thousands of DNA target sequences. However, there are also limitations to such MPRA studies. Our study was conducted in retinal explants from mouse models, which do not fully recapitulate human disease phenotypes. The identity of the TFs that bind CREs are not determined in MPRA studies, and many TFs have overlapping binding specificities. *Paired*-like homeodomain proteins recognize similar DNA motifs (Chu et al. 2012), and many homeodomain proteins are expressed in developing photoreceptors in addition to CRX and OTX2 (Markitantova and Simirskii 2020). These TFs likely act on a subset of CRX-bound CREs, and it is possible that some are dysregulated in the variant *Crx* mouse retinas. Other TFs may thus collectively contribute to altered CRE activities measured by MPRA. MPRAs do not reveal detailed biochemical mechanisms nor do they assess how CRE activities are affected by specific biochemical parameters, such as the kinetics and affinity of TF binding, as well as cooperative interactions between proteins. However detailed biochemical studies typically lack the scale to assess *cis*-regulatory mechanisms across hundreds or thousands of CREs with substantial differences in sequence composition. These differences in sequence composition likely affect biochemical parameters, and thus, large-scale assays of CRE activity are essential for understanding quantitative sequence-function relationships (Kinney and McCandlish 2019). Coding variants in TFs are linked with many human diseases (Tremblay et al. 2018; Herman et al. 2021; Sun and Chen 2023). To understand how different variants in the same TF gene produce different disease phenotypes, it is critical to understand their regulome-wide effects, which cannot be easily predicted from the biochemical details of TF–DNA and TF–TF interactions. Our work shows that MPRAs can yield insights into mechanisms of TF variants at different target CREs and complement the information learned from transcriptional and chromatin profiling assays.

## Methods

### Library design and cloning

The MPRA library contained 1723 genomic sequences centered on CRX ChIP-seq peaks that overlapped ATAC-seq peaks from P14 retinas (Corbo et al. 2010; Ruzycki et al. 2018). Sequences were 164 bp in length and represented in the library by four unique barcodes. We included 100 scrambled sequences as negative controls. Basal promoters were represented with 16 unique barcodes to ensure precise measurement of basal levels.

ChIP-seq peaks were sampled based on two sets of annotations previously described (Table 1; Ruzycki et al. 2018). These annotations include (1) the presence or absence of histone marks H3K4me3 and H3K27ac in P14 wild-type retinas and (2) the loss or retention of chromatin accessibility in *Crx*^−/−^ retina (i.e., whether accessibility is CRX dependent or independent). For each of the wild-type ChIP peaks in the library, we created a mutant version in which all copies of the CRX motif with a *P*-value of 2.5 × 10^−3^ or less were mutated by changing the core TAAT to TACT (White et al. 2013; Friedman et al. 2021). To test the hypothesis that CRX mutants might alter cooperative activity at CREs, we selected 100 library sequences with only two copies of the CRX motif and created single and double mutants.

The MPRA library was cloned following our previously described protocol (White et al. 2013, 2016; Friedman et al. 2021). Library sequences were synthesized by Agilent as barcoded oligonucleotides with the following structure: 5′ priming sequence (GTAGCGTCTGTCCGT)/EcoRI site/164-bp library sequence/SpeI site/C/SphI site/barcode sequence/NotI site/3′ priming sequence (CAACTACTACTACAG). The oligo library was amplified by four cycles of PCR using New England Biolabs (NEB) Phusion high-fidelity master mix. Amplicons were purified on a 2% agarose gel, digested with EcoRI-HF and NotI-HF (NEB), and then cloned into the EagI and EcoRI sites of the plasmid pJK03 with T4 ligase (NEB). The libraries were transformed into 5-alpha electrocompetent cells (NEB). The plasmid library was prepared by maxi-prep (Sigma-Aldrich), digested with SphI-HF and SpeI-HF (NEB), and then treated with Antarctic phosphatase (NEB). The *Rho* basal promoter with *DsRed* was amplified from pJK01 (Kwasnieski et al. 2012) and purified from a 1% agarose gel. The basal promoter amplicon was digested with NheI-HF and SphI-HF and then cloned into the libraries with T4 ligase (NEB). The library was transformed into 5-alpha electrocompetent cells (NEB), grown in liquid culture, and then prepared by maxi-prep (Sigma-Aldrich).

### Retinal explant electroporation and library sequencing

All mouse lines were on the C57BL/6J background and free of rd1 and rd8 mutations. The *R90W/+*, *R90W/W*, *E168d2/+*, and *E168d2/d2* lines were described by Tran et al. (2014). All procedures involving mice were approved by the animal studies committee of Washington University in St. Louis and performed under animal welfare assurance A-3381-01 and protocols 21-0414 (to S.C.). Experiments were performed in strict accordance with recommendations in the guide for the care and use of laboratory animals of the National Institutes of Health, the Washington University policy on the use of animals in research, and the guidelines for the use of animals in visual research of the Association for Research in Vision and Ophthalmology. Every effort was made to minimize the animals’ suffering, anxiety, and discomfort.

Retinal explant electroporations were conducted as previously described (Kwasnieski et al. 2012). Retinas were isolated from P0 mice and electroporated with 30 µg MPRA library and 30 µg pCAG-GFP as a transfection control. After culturing for 8 d, retinas were placed in TRIzol (Thermo Fisher Scientific), and RNA was extracted. Three replicate electroporations (three retinas each) were performed.

cDNA was prepared using SuperScript reverse transcriptase III (Thermo Fisher Scientific) with oligo(dT) primers following the manufacturer's instructions. Barcodes were amplified from cDNA or input plasmid DNA using 24 cycles of PCR with Phusion high-fidelity master mix (NEB). Amplicons were purified with the Monarch PCR kit (NEB), digested with MfeI-HF and SphI-HF (NEB), and ligated to custom sequencing adapters with PE2 indexing barcodes and phased P1 barcodes. Following 20 cycles of enrichment PCR, libraries were sequenced on the Illumina NextSeq platform to >1000× coverage per sample.

### MPRA quantification and analysis

All sequencing reads were processed regardless of quality score. Sequenced barcodes were matched to expected library members, and only reads with a sequenced barcode that perfectly matched an expected library barcode were counted. Barcode counts were normalized by reads per million (RPM) for each sample. To account for differences in barcode representation in the pooled library, RNA reads were normalized to input DNA reads (Kwasnieski et al. 2012). RNA/DNA ratios were averaged over all barcodes for each element and across biological replicates. CREs were classified into one of five activity groups based on their activity relative to the basal promoter alone. Expression levels were approximately log-normally distributed, so we computed the log-normal parameters for each sequence and then performed Welch's *t*-test. We corrected for multiple hypotheses using the Benjamini–Hochberg FDR procedure. We corrected for multiple hypotheses in each library separately to account for any potential batch effects between libraries. The log_2_ expression was calculated after adding a pseudocount of 1 × 10^−3^ to every sequence. CREs with a transcriptional activity that was not significantly different from basal were deemed “inactive.” Remaining CREs with an activity significantly greater than basal were deemed “strong enhancers” if their activity was greater than the 95th percentile of the 100 scrambled control sequences and were “weak enhancers” otherwise. “Strong silencers” and “weak silencers” were classified by having activity significantly lower than the basal promoter, with strong silencers less than the fifth percentile of the 100 scrambled controls. MPRA data for all library CREs are given in Supplemental Table S1. Metadata for each CRE are given in Supplemental Table S2. CRE sequences are given in FASTA format in Supplemental Data S1.

The information content and predicted CRX occupancy of each CRE sequence was computed as previously described (Friedman et al. 2021). Briefly, CRX predicted occupancy is determined by using the CRX energy weight matrix to calculate the probability of CRX binding at every 8-mer subsequence of each CRE. The binding probabilities at each position are summed to give the predicted CRX occupancy for the entire 164-bp sequence. Predicted occupancies for eight lineage-specific TFs described in the main text were used to calculate information content scores as described above and in work of Friedman et al. (2021).

To analyze motif enrichment across CRE subgroups, simple enrichment analysis (SEA; MEME Suite v5.5.2) was used with default settings and the HOCOMOCO Mouse v11 CORE motif database (Kulakovskiy et al. 2018; Bailey and Grant 2021). Analysis scripts and Python notebooks for the described analyses are available in the Supplemental Code S1 and at GitHub (https://github.com/barakcohenlab/retinopathy-manuscript). The completed MPRA analysis with activity and classifications of each CRE can be found in Supplemental Table S1.

## Data access

All raw and processed sequencing data generated in this study have been deposited at the NCBI Gene Expression Omnibus (GEO; https://www.ncbi.nlm.nih.gov/geo/) under accession number GSE230090.

## Supplementary Material

Supplement 1

Supplement 2

Supplement 3

Supplement 4

Supplement 5
