# Supplementary material for: Pathogenic variants in CRX have distinct cis-regulatory effects on enhancers and silencers in photoreceptors
Source: Genome Res. 2024 Feb;34(2):243–55. doi: 10.1101/gr.278133.123 (PMC10984388; doi:10.1101/gr.278133.123)
Supplement: Supplement 1 [file Supplemental_Figures.pdf]

Supplementary Figures

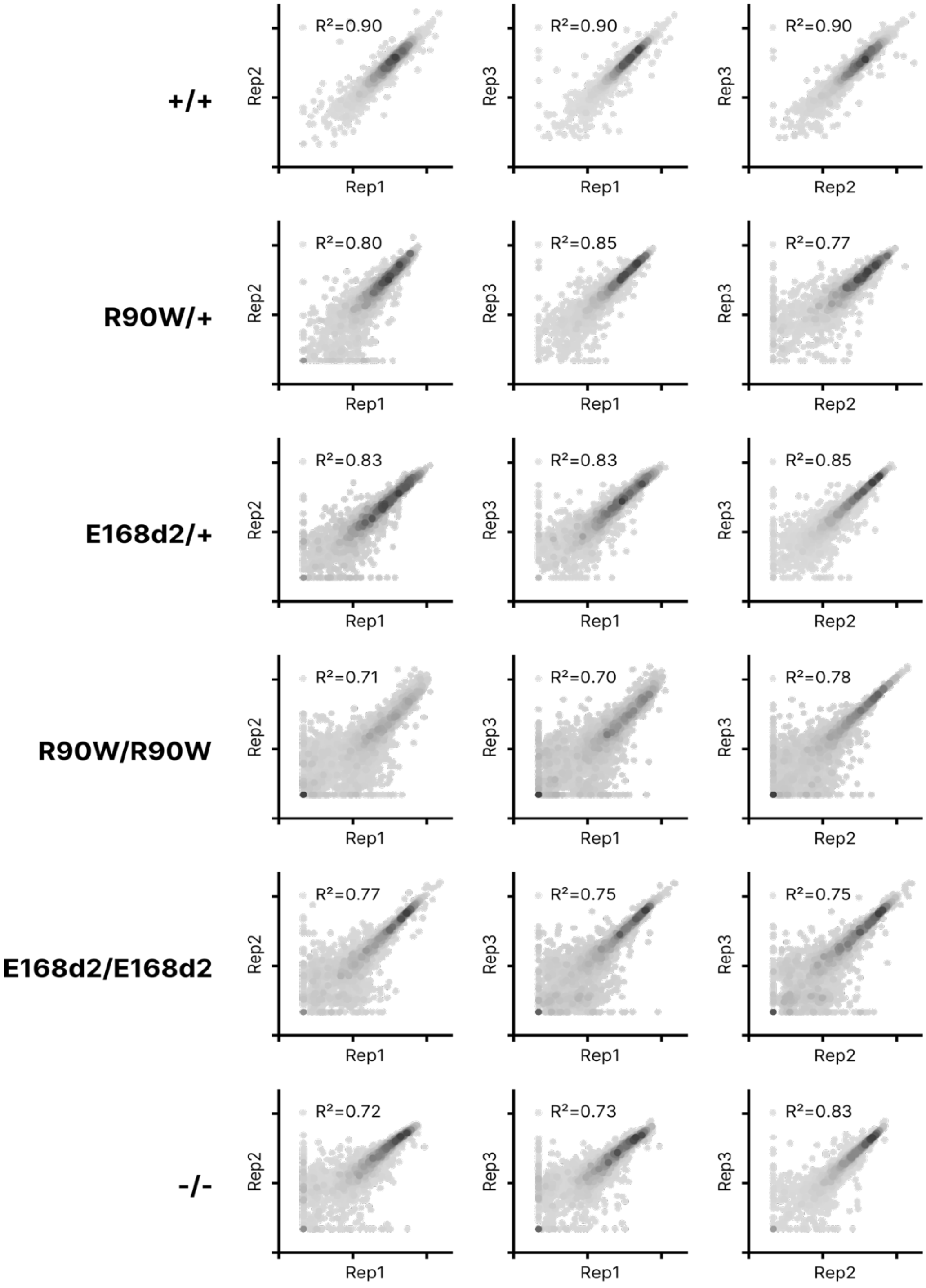

Figure S1. CRE-level correlation across replicates for the *Rho* promoter library for the indicated genotypes.

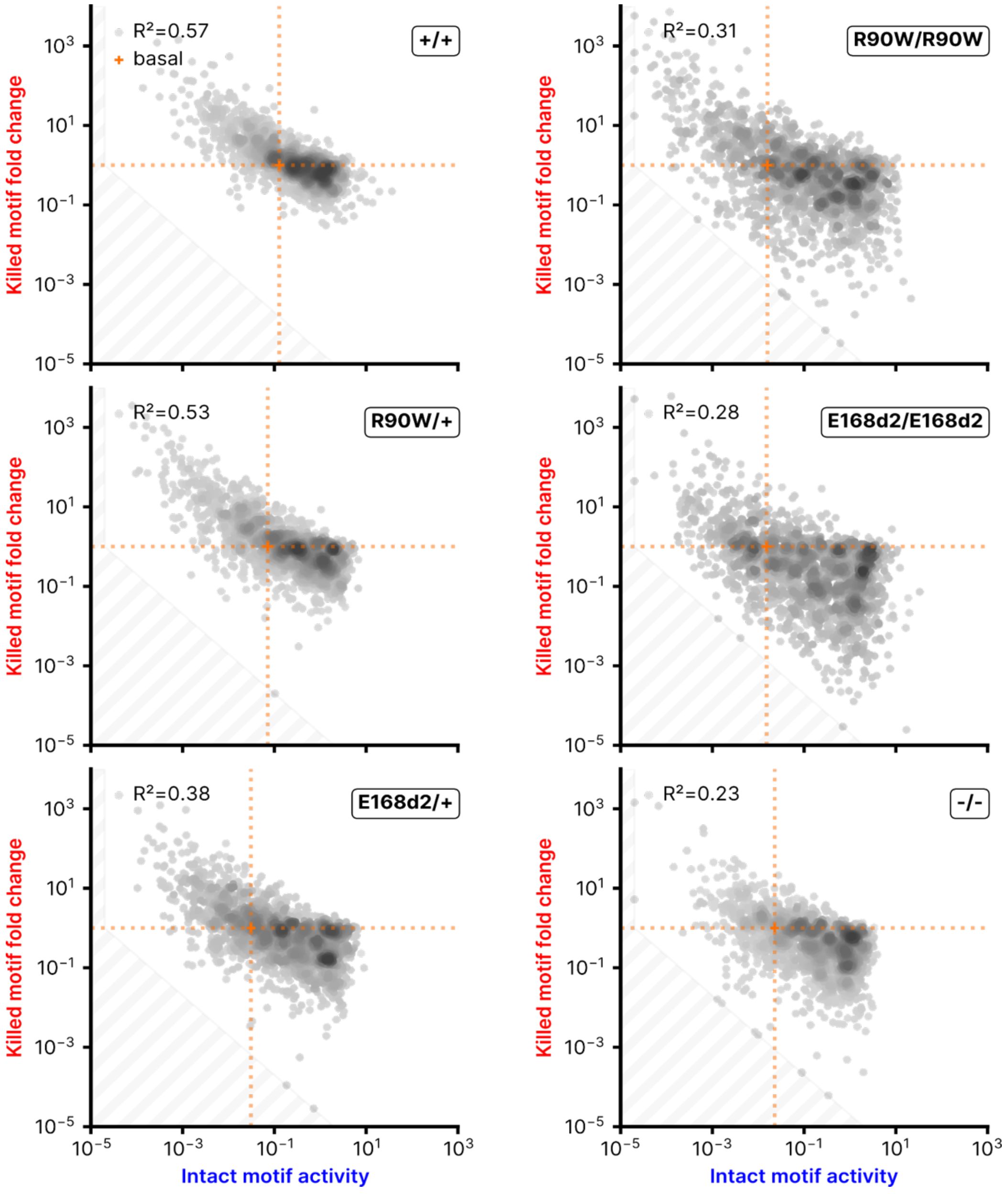

Figure S2. Activity of each CRE with intact CRX motifs versus the fold change in activity for each matching CRE with killed motifs, in each of the indicated genotypes. The orange dotted lines indicate the activity of the basal promoter alone, and a fold change of 1 (no difference in activity between CRE with intact or killed CRX motifs). In all panels, transcriptional activity was adjusted for visualization purposes using a fixed pseudocount of 2×10^{-5}; the out-of-bound region is indicated by the light gray stripes.

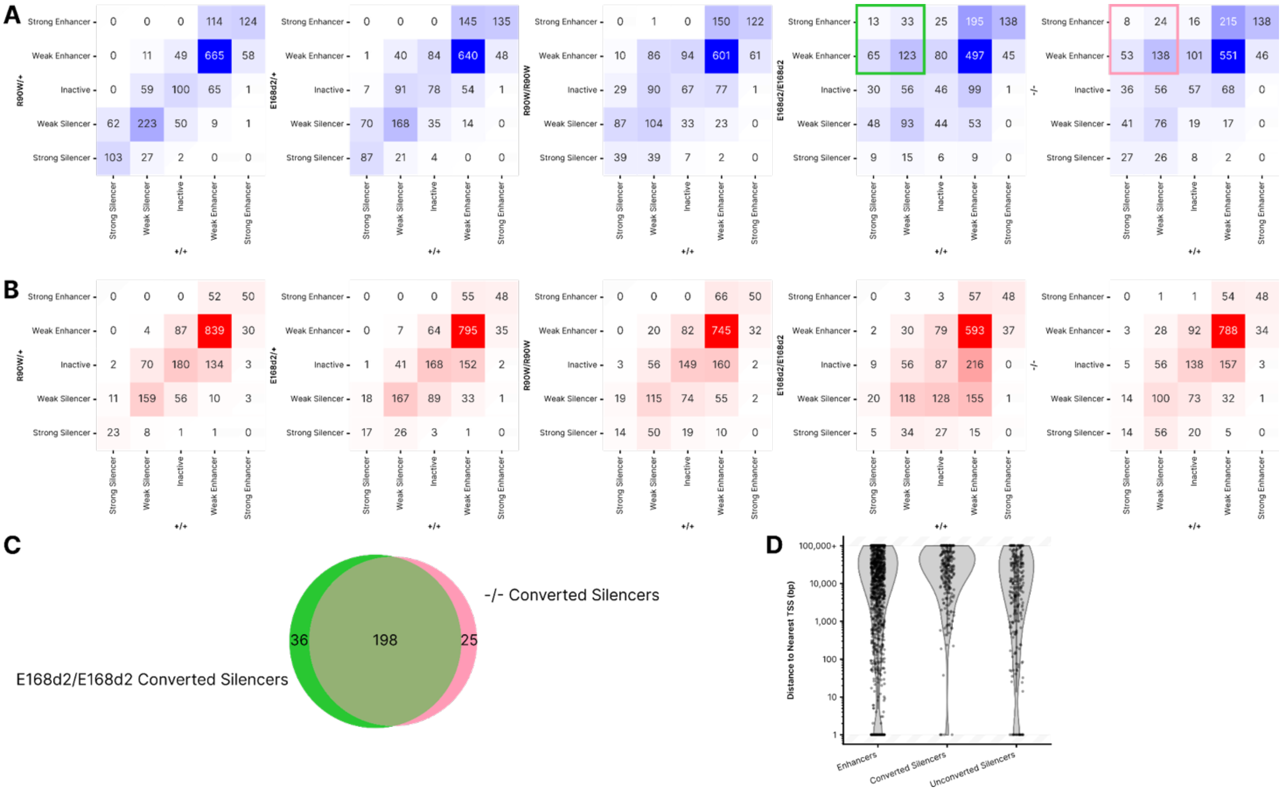

Figure S3. Activity classifications of CREs across *Crx* genotypes. (**A**) Classifications of all CREs with intact CRX motifs. (**B**) Classifications of all CREs with killed CRX motifs. (**C**) Overlap of CREs classified as silencers in +/+ and enhancers in the indicated genotypes; colors match the boxes in panel A. (D) Distances from the CREs in the indicated subgroups to the nearest transcriptional start site. Distances were clipped to 100 kbp.

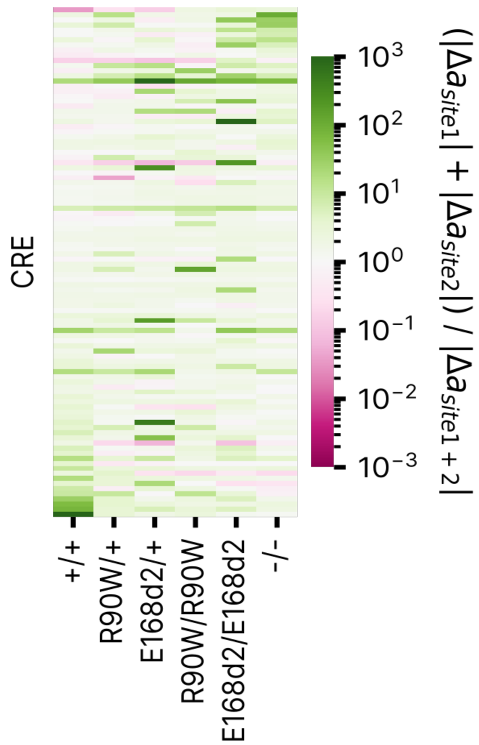

Figure S4. Analysis of cooperativity between CRX sites across *Crx* genotypes. Heatmap of the ratio of the sum of single mutant transcriptional activity delta to the double mutant transcriptional activity delta; each row is a different CRE. CREs are sorted by the ratio of the cooperativity score in +/+ and -/- genotypes.
